# Financial Status, Burden, and Family Caregiver Well-Being: A Cross-Sectional Analysis

**DOI:** 10.1093/geroni/igaf122.3936

**Published:** 2025-12-31

**Authors:** Megan Thomas Hebdon, Amy Patten, Sandi Alshahwan, Carolyn Phillips

**Affiliations:** The University of Utah, Salt Lake City, Utah, United States; University of Texas at Austin, Austin, Texas, United States; University of Utah, Salt Lake City, Utah, United States; University of Texas at Austin, Austin, Texas, United States

## Abstract

Family caregivers are fundamental to the care and support of individuals with chronic illnesses and disabilities. Both caregiver burden and financial challenges may impact the health and well-being of family caregivers. The purpose of this study was to explore the impact of financial status and caregiver burden on caregiver well-being. Family caregivers (N = 60) of individuals with chronic illnesses and/or disabilities were recruited using online recruitment methods. A rigorous fraud protocol was used to screen potential participants. After screening and consent procedures, participants completed an online cross-sectional survey with demographic questions, including income and perceived financial adequacy, and measures for caregiver burden and holistic well-being. Data were analyzed using descriptive statistics, correlation analysis, and hierarchical linear regression. Caregivers were primarily women (n = 48; 80%) and White (n = 40; 67%). Half reported having an adequate income (n = 30; 50%; average income $58,873.25, SD = 41,187.05). Annual income was significantly associated with financial adequacy (r=.42, p<.01) and meaning and purpose (r=.28, p < 01), but no other well-being domains. Annual income and perceived financial adequacy were not significantly related to caregiver burden. Perceived financial adequacy was significantly positively associated with all well-being domains (r=.30-.47, p<.01,). Caregiver burden was significantly negatively associated with all well-being domains, except financial and material stability (r=-.23, p=ns). When controlling for income, the relationships among financial adequacy, caregiver burden, and well-being domains remained. Our findings suggest that caregivers’ perceptions of their financial situation have more impact on well-being than income. In addition, burden and perceived financial adequacy may be key independent factors related to caregiver well-being.

